# The use of a percutaneous cystostomy tube as an adjunctive treatment option for dogs with idiopathic functional outflow tract obstruction

**DOI:** 10.1111/jvim.17275

**Published:** 2024-12-19

**Authors:** Zoe P. Greenfield, Allyson C. Berent, Chick W. Weisse

**Affiliations:** ^1^ Schwarzman Animal Medical Center, 510 East 62nd Street New York, New York 10065‐8314 USA; ^2^ Schwarzman Animal Medical Center, 510 East 62nd Street New York, New York 10065 USA; ^3^ Schwarzman Animal Medical Center, Diagnostic and Interventional Radiology, 510 East 62nd Street New York, New York 10065 USA

**Keywords:** cystostomy tube, detrusor urethral dyssynergia, functional outflow tract obstruction, urethral obstruction

## Abstract

**Background:**

Functional outflow tract obstruction (FOO) remains a challenging disease to manage in male dogs. Cystostomy tubes have been used to relieve urethral obstruction while allowing time to achieve effective medical management, avoiding the need for emergency visits and repeat urinary catheterizations.

**Objectives:**

To describe a series of dogs with FOO and categorize the most successful management strategies including medical management alone or with the support of cystostomy tubes for urinary diversion.

**Animals:**

Twelve client‐owned dogs with FOO were included.

**Methods:**

Medical records of dogs presented from August 2012 to September 2021 with a presumptive diagnosis of FOO were retrospectively reviewed and findings recorded.

**Results:**

Seven dogs were managed with a cystostomy tube and 5/7 (71%) had a good‐excellent outcome. Five dogs were managed without a tube and 1/5 (20%) had a good outcome. Eight dogs were refractory to medical management; 5 managed with a cystostomy tube and 3/5 (60%) had a good‐excellent outcome; 3 managed without a cystostomy tube and 0/3 had a good or excellent outcome. Overall, dogs with cystostomy tubes had better outcomes but also had mild, moderate, and severe complications (100%, 71%, and 71%, respectively) reported.

**Conclusions and Clinical Importance:**

Most dogs that received a percutaneous cystostomy tube as part of their management plan had a good to excellent outcome, particularly when compared to those that did not, despite the high rate of tube‐related complications. Percutaneous cystostomy tubes could be considered early during management for FOO to improve overall outcomes.

**Abbreviations**
ACVIM: American College of Veterinary Internal Medicine
AMC: The Schwarzman Animal Medical Center
AST: aspartate aminotransferase
AUS: abdominal ultrasound
BUN: blood urea nitrogen
CK: creatine kinase
DRD: detrusor reflex dyssynergia
EMG: electromyography
FOO: functional outflow tract obstruction
MOO: mechanical outflow tract obstruction
MRI: magnetic resonance imaging
PVRV: post‐void residual volume
SD: standard deviation
UTI: urinary tract infection

## 1 INTRODUCTION

Idiopathic urinary functional outflow obstruction (FOO) refers to a voiding disorder where the bladder fails to contract, the urethra fails to relax, or a combination of both, resulting in a large post‐void residual volume (PVRV) and an obstructive pattern of urination without a mechanical obstructive lesion detected. In veterinary medicine, FOO has been called a variety of names including detrusor urethral dyssynergia, reflex dyssynergia, and idiopathic functional urinary outflow tract obstruction. In 2024, the American College of Veterinary Internal Medicine (ACVIM) Consensus Statement on urinary incontinence adopted the term FOO to globally characterize this non‐neurogenic functional voiding disorder.^1^

Functional outflow obstruction typically affects young to middle‐aged male dogs of large/giant breeds.^2^, ^3^, ^4^ Few reports have suggested female dogs may rarely be affected by this condition, but full diagnostics were not defined to ensure that spinal cord injury, or another underlying disease, were not the cause.^3^, ^4^ Generally, a diagnosis of FOO is a diagnosis made after ruling out a mechanical outflow obstruction (MOO) or neurological abnormality. Imaging modalities utilized in previous reports have included abdominal ultrasound (AUS), computed tomography (CT), magnetic resonance imaging (MRI), cystoscopy, and contrast cystourethrography.^2^, ^3^, ^4^ Urodynamic testing is not commonplace in veterinary medicine largely owing to the lack of standardization of equipment and anesthetic protocols as well as equipment availability, male dog anatomy, and expense and pursuing conscious/non‐anesthetized testing such as uroflowmetry is not feasible in unlike in humans.^5^, ^6^, ^7^, ^8^, ^9^, ^10^

Medical management standardly includes an alpha‐adrenergic antagonist and often a skeletal muscle relaxant. The addition of parasympathomimetics, anti‐inflammatories, or anxiolytics is clinician dependent.^1^, ^2^, ^3^, ^4^ Outcomes are variable with some dogs responding to single agent therapy and some requiring chronic at home urinary catheterizations. When medical management fails, other interventions for this condition reported included castration, indwelling urinary catheters, urethral stenting, urethral balloon dilation, and cystostomy tubes.^4^, ^11^, ^12^, ^13^ There are limited reports on the use of cystostomy tubes as a management strategy for FOO and their overall contribution to successful medical management long‐term.

In human medicine, the disease counterpart is detrusor reflex dyssynergia (DRD).^4^, ^14^ In contrast to veterinary medicine, this is a neurogenic voiding disorder and diagnosis is typically based on electromyography (EMG) recordings, voiding cystourethrogram, and urodynamic studies. Treatment options include pharmacotherapy with alpha blockers and skeletal muscle relaxants (ie, tamsulosin and diazepam), clean intermittent self‐catheterization, transurethral botulinum injections, and urinary sphincterotomy.^15^, ^16^, ^17^

Reports of FOO have limited cases managed with a cystostomy tube.^4^, ^14^ The largest retrospective study evaluating cystostomy tubes and associated complications in veterinary medicine did not include any cases with FOO.^11^ Moreover, these tubes were placed surgically whereas percutaneous placement is now more commonplace, especially for temporary and acute use. In the authors experience, the use of a temporary percutaneous cystostomy tube has been well received by clients, allowing them time to adjust medical therapies while serving as a non‐traumatic means of at home urinary drainage, testing pharmacologic interventions with careful monitoring of micturition, while avoiding further urethral stimulation with indwelling or intermittent urethral catheterization. In addition, maintaining the bladder in a non‐distended state reduces the risk of secondary detrusor atony which can be irreversible if left untreated.^1^

The objective of this study was to describe a series of dogs with FOO and categorize management strategies at 1 institution including medical management alone or medical management with the support of a cystostomy tube.

## 2 MATERIALS AND METHODS

### 2.1 Case selection

Medical records of male dogs presented to The Schwarzman Animal Medical Center from August 2012 to September 2021 were searched electronically using the keywords “reflex dyssynergia,” “detrusor urethral dyssynergia,” “functional urinary/urethral obstruction,” and “benign urethral obstruction.” Inclusion criteria required documented clinical signs consistent with FOO including stranguria and dysuria with a lack of a structural abnormality documented on imaging. In addition to AUS or abdominal radiographs, all dogs were required to have either cystourethroscopy or contrast cystourethrography performed to enable evaluation of the urethra and to rule‐out a MOO. Dogs were excluded if they had documented abnormalities causing a mechanical outflow tract obstruction (eg, urethral stricture, cystoltihs, urethroliths, proliferative urethritis, a urethral or bladder mass, had previous urethral surgery, etc.) or concurrent abnormal neurologic findings on examination. A documented urinary tract infection (UTI) at the time of diagnosis did not exclude dogs unless urinary signs were completely resolved with documentation of clearing of the infection. A minimum follow‐up time of at least 30 days was required for inclusion. Follow‐up included client communications, referring veterinarian records, or formal recheck examinations to document status of urination, ability to empty bladder, UTIs, tube patency, and urination quality.

### 2.2 Medical record review

Data were collected including signalment, weight, neuter status, presenting clinical signs, physical examination findings (including a neurologic examination), referral diagnostic testing, treatment history, intake diagnostic testing, urine microbiological results, imaging results, management strategies and medication dosages, complications, overall outcomes, and follow‐up time.

### 2.3 Placement of the percutaneous cystostomy tube

**FIGURE 1:**
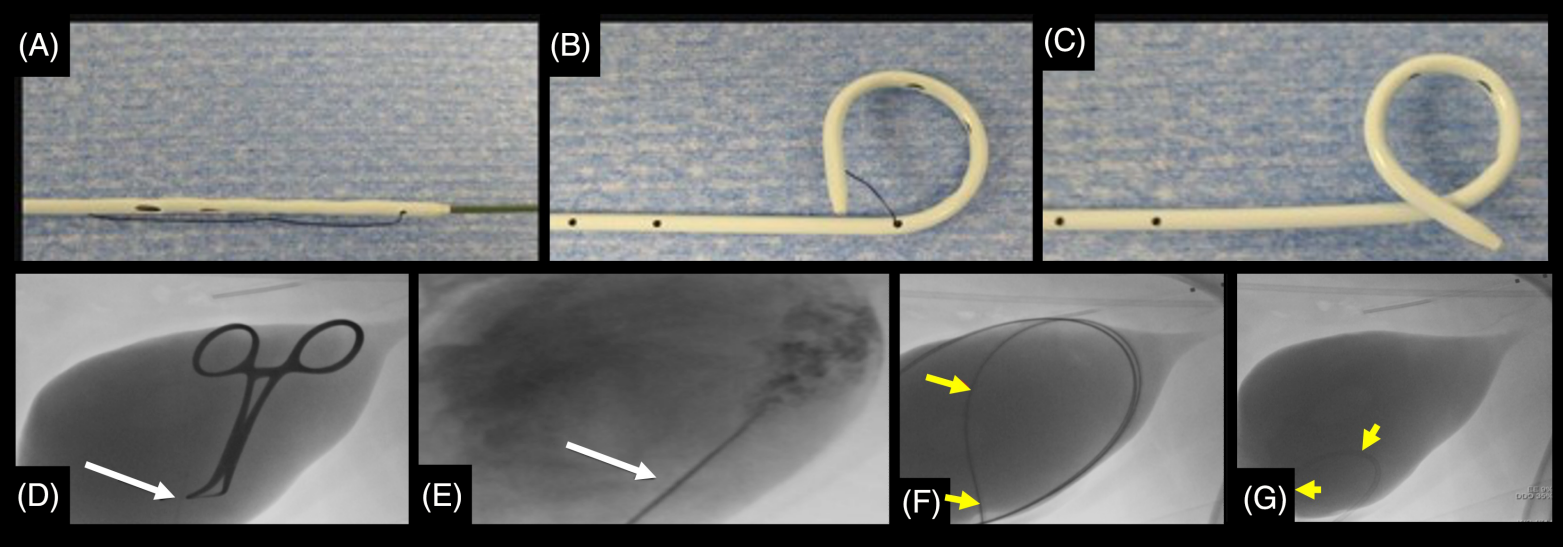
Images of a 6Fr. locking‐loop pigtail catheter (A‐C) and fluoroscopy images during percutaneous cystostomy tube placement (D‐G). (A) The distal end of the locking‐loop pigtail catheter over the metal stylette and guidewire; notice the multiple fenestrations and locking string. (B) After the stylette and guidewire are withdrawn, notice the pigtail shape forming as the string is tightening. (C) The string is being locked, creating a tight curl in the distal end of the catheter. (D) White arrow indicates an 18‐ga. intravenous catheter establishing access to the urinary bladder for cystocentesis. (E) The intravenous catheter is further advanced allowing for a contrast cystogram. (F) A guidewire (yellow arrows) is advanced within the urinary bladder and the locking‐loop pigtail is advanced over the guidewire into the urinary bladder. (G) The guidewire is removed and the pigtail catheter (yellow arrows) is locked within the urinary bladder and retracted toward the bladder wall.

The percutaneous cystostomy tube was placed using a standard technique described elsewhere.^18^ In short, dogs were placed under general anesthesia in lateral recumbency and the abdomen was standardly prepared. The urinary bladder was isolated with manual palpation or ultrasound, and with the aid of fluoroscopy, an intravenous catheter was used for cystocentesis, followed by a contrast cystogram. Next, a guidewire was advanced into the urethral bladder, out the urethra. Over the guidewire a locking‐loop pigtail catheter (Infiniti Medical, Redwood City, California) was placed into the urinary bladder and locked in place. The catheter was secured to the body wall using a purse‐string suture and finger‐trap pattern and placement was successfully confirmed using fluoroscopy and contrast. See Figure 1.

### 2.4 Categorization

Outcomes were categorized as excellent, good, fair, and poor as detailed in Table 1. The authors reviewed methods of evaluating and scoring complications utilized previously in the veterinary literature and adapted 1 for dogs with cystostomy tubes as detailed in Table 2.^19^, ^20^ Dogs were defined as refractory or non‐refractory to medical management. A refractory case was defined as a dog with a poor response to appropriate medical therapies of at least 1 week duration resulting in recurrent episodes of outflow tract obstruction, exclusive use of the cystostomy tube, or consideration of euthanasia because of repeat obstructive episodes or quality of life concerns. In this study, for a dog to be considered refractory, the use of an alpha‐adrenergic antagonist and a skeletal muscle relaxant, concurrently, at recommended doses based on the ACVIM Consensus statement, was required, for at least 1 week duration.^1^ The standard approach to medical management, as outlined by the ACVIM consensus statement, consists of an alpha‐adrenergic antagonist in a recommended dose range (tamsulosin at 0.4‐0.8 mg/dog PO q24h or prazosin 0.5‐3 mg/dog PO q8‐12h). In the statement, not all authors' considered a skeletal muscle relaxant as a first line agent, but was generally used if a primary alpha‐adrenergic antagonist failed.^1^ For this reason, both were required for the authors to consider a dog refractory.

**TABLE 1:** Outcomes for dogs with FOO, with and without a cystostomy tube.

Outcome	Definition
Excellent	Demonstrated resolution of signs with no chronic urinary medications and no episodes requiring medical management or further intervention (ie, urinary catheterization).
Good	Demonstrated resolution of signs with medical therapy or if flare‐ups these were able to be managed medically without need for urinary catheterization for an obstructive episode. If a cystostomy tube was placed, it was able to be removed and did not require replacement in the future.
Fair	Demonstrated resolution of signs with medical therapy but had recurrent urethral obstruction requiring a veterinary visit and/or catheterizations. Alternatively, if a cystostomy tube was in place, it was used intermittently, exclusively, or removed and required replacement.
Poor	Defined as a dog who was unable to urinate normally despite medical therapy, resulting in at home urinary catheterizations, had severe tube complications resulting in death or euthanasia, or euthanasia secondary to FOO over quality‐of‐life concerns.

**TABLE 2:** Complications associated with percutaneous cystostomy tubes.

Complication	Definition
Mild	Those that resolve or respond to select medical treatment (antiemetics, antipyretics, analgesics, diuretics, and electrolytes) or bed‐side procedures without need for tube replacement. Examples include irritation or inflammation around the stoma site, urine leakage around the tube, hematuria, tube obstruction, an isolated urinary tract infection, suture breakage.
Moderate	Those that require more extensive drug use or the need for a simple intervention. Examples include breaking and/or chewing of the tube, the need for tube exchange/or replacement through an established tract or with guide wire tube exchange, chronic infections, uroperitoneum requiring hospitalization for medical management without the need for surgical repair.
Severe	Those that require a more complicated intervention under general anesthesia, or a complication that resulted in death or euthanasia of the dog because of the complication. Therefore, inadvertent tube removal was only classified as a severe complication if immediate replacement was required necessitating a new stoma to be made or surgical intervention, or it resulted in a uroperitoneum requiring surgical intervention. Otherwise, inadvertent tube removal without need for immediate replacement (within 1 month), was classified as a moderate complication.

A UTI was defined as a positive urine culture with concurrent pyuria and clinical signs including hematuria and malodorous urine.

### 2.5 Statistical analysis

Descriptive statistics were utilized in this study using Microsoft Excel (2018). Most data were summarized using mean, median, and range. Frequency analysis provided frequencies for categorical variables and the percentage contribution to the entire population for that variable.

## 3 RESULTS

### 3.1 Case selection

Sixteen male dogs diagnosed with FOO met the inclusion criteria. Four of these dogs were excluded owing to lack of follow‐up data resulting in a total of 12 male dogs. Breeds identified included Labrador Retriever (3), Akita (2), German Shepherd (2), Cane Corso (1), Chesapeake Bay Retriever (1), Golden Retriever (1), mixed breed dog (1), and Schnauzer (1). Six dogs were intact and 6 neutered at the time of diagnosis. The dogs ranged from 1.1 to 11.4 years (median, 7.5). Initial clinical signs noted in the dogs' medical records included stranguria (12), urinary obstruction on presentation or within 1 week of referral (11), pollakiuria (6), and vomiting (4). Initial signs consistent with FOO preceded evaluation by a mean of 273 days and median of 270 days (range, 1‐1095). Six of 12 dogs (50%) were referred by their primary veterinarian and for all others, the clients sought specialty care on their own. Two dogs were neutered with the primary veterinarian in conjunction with clinical signs consistent with FOO and 6 of 12 (50%) were started on medical management with the primary veterinarian. Of these 6 dogs, the most common drug prescribed was prazosin in 5 of 6 at a median dose of 0.04 mg/kg PO q8‐12h (range, 0.02‐0.07 mg/kg PO q12h), followed by tamsulosin in 2 of 6 at a median dose of 0.015 mg/kg PO q12h (range, 0.012‐0.017 mg/kg PO q12h). One dog was prescribed bethanechol at a dose of 0.3 mg/kg PO q12h and no dogs were prescribed diazepam. Five of 6 dogs were prescribed an antibiotic although only 2 of 5 had urine testing performed, neither of which were consistent with a UTI.

### 3.2 Medical record review

A complete blood count (CBC) and serum biochemistry profile were available to review for 10 of 12 dogs. Abnormalities on CBC were uncommon with a neutrophilia in 2/10 (mean 8.02 K/μL, median 7.3, range, 3.84‐14, reference range, 2.94‐12.67 K/μL) and an anemia in 2/10 (mean Hct 45.1%, median 46.7%, range, 35%‐50.7%, reference range, 38.3%‐56.5%). All 10 dogs had normal creatinine and 2/10 had an elevated blood urea nitrogen (BUN) (mean 19.3 mg/dL, median 15.5, range, 9‐38, reference range, 9‐31 mg/dL). The most common hematologic abnormalities identified were an abnormally high aspartate aminotransferase (AST) and creatine kinase (CK) activities in 4/10 and 4/10 respectively (mean 81.8 U/L, median 34.5, range, 20‐361, reference range, 16‐55 U/L; mean 339.8 U/L, median 172.5, range, 56‐1299, reference range, 10‐200 U/L, respectively). Hypoalbuminemia was identified in 3/10 (mean 2.97 g/dL, median 3.05, range, 2.2‐3.7, reference range, 2.7‐3.9 g/dL). Urinalysis was available for review in 10/12 dogs and a urine microbiological culture in 12/12. The urinalysis showed bacteriuria in 1/10, pyuria in 7/10, and microhematuria in 9/10. Urine culture was positive in 2/12; with *Pseudomonas aeruginosa* and *Escherichia coli*, respectively.

Imaging was performed in all dogs including an AUS (11/12), abdominal radiographs (10/12), contrast cystourethrography (8/12), and cystourethroscopy (7/12). The most common abnormalities noted on AUS included a diffusely thickened bladder wall consistent with cystitis (n = 6) and a symmetrically enlarged prostate consistent with benign prostatic hyperplasia (n = 4). Three dogs had a normal AUS. The only finding on abdominal radiographs was prostatomegaly (n = 3). Findings on contrast cystourethrography included a narrowing in the pelvic urethra (n = 2) consistent with a normal prostatic urethra. Findings on cystourethroscopy included erythema of the bladder wall (n = 5), fibrinous strands in the urethra (n = 4) consistent with prior trauma from catheterizations, and smooth, symmetric, aligned lesions that looked as if formed by contact and friction across folds in the urethra (n = 3).

### 3.3 Management

Medical management was attempted as an initial means to control FOO in 11 of 12 dogs, 6 of which were started on therapies by the primary veterinarian. Of the 11 dogs that were originally managed medically, they were managed with an alpha‐adrenergic antagonist including tamsulosin (n = 6; mean 0.019 mg/kg PO q12‐24h) or prazosin (n = 5; 0.05 mg/kg PO q12h), diazepam (n = 2; 0.2 mg/kg PO q8h), and bethanechol (n = 2; 0.18 mg/kg PO q12h). Eight of 12 dogs received treatment in addition to pharmacotherapy including a cystostomy tube (n = 7) and neuter (n = 5). Surgery, either neuter and/or cystostomy tube placement, was pursued 162 days (mean; median 21, range, 4‐548) following initial clinical signs consistent with FOO. Of the 12 male dogs in this study, 6 were neutered and 6 intact at the time of initial diagnosis. Of the 6 intact dogs, 5 (83%) were neutered in conjunction with their diagnosis. Four of 5 dogs that were neutered had a cystostomy tube placed concurrently with the neuter (n = 1) or following the neuter (n = 3); only 1 dog was neutered as a sole adjunctive therapy to medical management. Two dogs were neutered before referral and 1 of these dogs had no adjunctive medical therapy; this dog presented through emergency with a urinary obstruction.

Eight of 12 (67%) dogs were categorized as refractory to medical management; 5 were managed with a cystostomy tube, 2 continued medical management, and 1 was having at‐home intermittent urinary catheterizations. The remaining 4 dogs were non‐refractory; 2 were managed with a cystostomy tube and medical management and 2 solely with medical management.

### 3.4 Cystostomy tubes

Seven dogs were managed with a cystostomy tube, 5 of which were refractory to medical management. Three dogs were defined as refractory to medical management before cystostomy tube placement and had their tubes placed a mean of 59 days following initiation of medical management (range, 8‐153 days). Four dogs had cystostomy tubes placed before optimal medical management; 2 responded to optimal medical management and 2 were refractory. All dogs managed with a cystostomy tube were also concurrently being maximally medically managed. All dogs were managed with diazepam (n = 7, median 0.33 mg/kg PO q8h) and an alpha‐adrenergic antagonist such as tamsulosin (n = 6, median 0.009 mg/kg PO q12h) or prazosin (n = 1, 0.07 mg/kg q8h). Four dogs received gabapentin and 2 received a non‐steroidal anti‐inflammatory. Five of 7 dogs achieved complete disuse of the cystostomy tube documented at 1‐2 weeks (n = 2), 3‐4 weeks (n = 1), >4 weeks (n = 2) post placement. One dog in this study was euthanized within 30 days of tube placement and owners were exclusively using the tube throughout this time with a failed response to maximized medical management.

There were 15 tubes placed across 7 dogs; mean 2.14 tubes per dog (median 2, range, 1‐5). Fourteen of 15 tubes were placed percutaneously as described above. The dog that had a surgically placed cystostomy tube had 5 tubes, the third of which was surgically placed. The condition of cystostomy tube removal was evaluated for each tube; 5/15 (33%) tubes were removed by the clinician because of improvement, 5/15 (33%) were removed by the dog and not replaced because of improvement, 4/15 (27%) were removed by the dog and immediately replaced given continued tube dependence, and 1 was removed by the dog and not replaced as euthanasia was pursued.

### 3.5 Outcomes

Ten of 12 dogs (83%) had documented improvement in their urination; 7 improved within 1‐2 weeks while 3 took greater than 4 weeks. Of the 2 dogs that had no improvement, 1 was managed with a cystostomy tube and 1 with medical management alone. Overall outcomes for this study were excellent (n = 1), good (n = 5), fair (n = 3), and poor (n = 3). A majority of dogs managed with a cystostomy tube had a good to excellent outcome (5/7, 71%) compared to those managed without a cystostomy tube where fair or poor outcome was most common in 2/5 and 2/5, respectively (40% fair and poor). Eight of 12 dogs were refractory to medical management; 5 were managed with a cystostomy tube and 3/5 (60%) had a good to excellent outcome; 3 were managed with medical therapy and at‐home routine catheterization (n = 1) or medical management only (n = 2), and none of these cases were classified as having a good to excellent outcome.

### 3.6 Follow‐up

Follow‐up data was available for all dogs in this study and ranged from 40 to 1671 days. The median follow‐up time for all dogs was 187 days; for dogs with a cystostomy tube the median follow‐up time was 323 days (range, 48‐895) and for dogs without a cystostomy tube it was 128 days (range, 40‐1671). Four dogs in this study were euthanized; 3 for reasons unrelated to FOO including metastatic neoplasia (n = 2) and a spontaneous hemoabdomen and hemothorax (n = 1). Euthanasia secondary to FOO was only pursued in 1 dog. This dog was refractory to medical management and removed the cystostomy tube on their own. This dog had a UTI and was hospitalized for urinary monitoring after removal and developed signs of sepsis, resulting in the owner electing euthanasia. The cause of sepsis in this dog was suspected to be because of the UTI but was unassociated with any evidence of a uroabdomen or recurrence of the urinary tract obstruction, therefore unlikely a tube related complication.

### 3.7 Recurrence of obstruction

Only 1 dog that was managed with a cystostomy tube had a recurrence of urinary obstruction following removal; this occurred 180 days after tube removal. Recurrent urinary obstruction for those exclusively medically managed was more common and occurred in 4/5 dogs (80%). Of note, the only dog without recurrent urinary obstruction in this group had the shortest follow‐up time of 40 days compared to a mean of 421.8 days (range, 40‐1671).

### 3.8 Cystostomy tube associated complications

Tube complications were categorized as either mild, moderate, or severe as outlined above. Of the 7 dogs managed with a cystostomy tube, 7/7 (100%) had mild complications including loose suture (n = 5), leaking around the stoma (n = 4), and stoma inflammation (n = 3). Five of 7 (71%) dogs were considered to have moderate complications including inadvertent tube removal by the dog which did not require replacement (n = 3), and chronic UTIs as defined above (n = 3). Severe complications occurred in 5 of 7 (71%) dogs including tube dislodgment requiring immediate replacement (n = 3) and leaking of urine around the tube resulting in a uroabdomen requiring a tube exchange (n = 2).

## 4 DISCUSSION

This retrospective study reviews a large number of cases utilizing percutaneous cystostomy tubes for the management of FOO, particularly focusing on FOO refractory to medical management alone. Overall, dogs with cystostomy tubes had a good to excellent outcome regardless of whether they were refractory to medical management, or had complications related to their tubes, and this appeared to be superior to the group of dogs that did not have a cystostomy tube as part of their management plan.

Retrospective evaluation of these 12 cases demonstrates a majority of dogs with FOO managed with a cystostomy tube had an excellent to good outcome (5/7; 71%) compared to those that were only medically managed, without a cystostomy tube. Dogs managed without a tube had no excellent outcomes and only 1 (20%) was classified as a good outcome. A majority of cases evaluated in this series would be considered medically refractory (8/12; 5/8 managed with a cystostomy tube and 3/8 managed medically), and 60% of refractory dogs had a good to excellent outcome when treated with a cystostomy tube compared to 0 dogs when not managed with a cystostomy tube.

There was an over‐representation of large breed middle‐aged dogs of 7.2 years (mean) in this study similar to that previously reported.^2^, ^3^, ^4^ However, several owners reported consistent signs up to 3 years before diagnosis or management. FOO has a range of severity from pollakiuria, stranguria and partial obstruction to overt urethral outflow obstruction. Therefore, the true age of onset for this disease is not clear.

Of the 12 male dogs in this study, 6 were neutered and 6 intact at time of diagnosis. Of the 6 intact, 5 were neutered throughout the study. Three of these dogs required a cystostomy tube because of persistent signs following the neuter. One dog was neutered as a sole means of therapy and presented with a urinary obstruction on referral 300 days after the neuter. Only 1 dog remained intact after their diagnosis, and this dog was managed on finasteride, tamsulosin, and diazepam. These findings support that although neutering may be beneficial in these cases, it is an ineffective treatment without concurrent pharmacotherapy and dogs still may require a cystostomy tube to get to clinical stability.

Antibiotics were commonly prescribed to this group of dogs with their initial signs of FOO. Of the 6 dogs receiving some sort of medical treatment for their clinical signs before specialty care, 5 (83%) were prescribed antibiotics, yet none of these dogs had urinalysis or urine culture findings to support utilization of antibiotics. Generally, UTIs are not associated with outflow tract obstructions, but may present with dysuria and hematuria. Urine testing is recommended in all dogs with lower urinary tract signs before consideration of antimicrobials, and alternatives to infections should be considered, especially in male dogs.

We suspected that cystostomy tubes would reduce the number of overall visits to the hospital for obstructions, given the owners ability to void a dog's bladder in an emergency situation. No dogs with a cystostomy tube were evaluated for a urinary obstruction while the tube remained in place compared to 4 of 5 dogs (80%) that were exclusively medically managed. Moreover, only 1 dog that was managed with a cystostomy tube had a recurrent urinary obstruction and this occurred 180 days following removal of the tube.

Complications associated with the cystostomy tube were common with all dogs having mild complications and 5 of 7 having moderate and severe complications. Complications are comparable to previous studies with cystostomy tubes and that includes suture loosening, leaking around stoma, and tube dislodgement.^11^, ^20^ Inadvertent tube removal occurred in 6 dogs; 3 dogs with inadvertent tube removal had no further intervention and 3 required immediate tube replacement to continue FOO management. Only 2 dogs required an intervention over concern for a uroabdomen, neither of which resulted from inadvertent tube removal.

This study might support that percutaneous cystostomy tubes could be considered early in the course of management for FOO to avoid recurrent obstructive episodes or at home intermittent catheterization, which may avoid premature euthanasia. Tubes allow time and patience to find the right medical regimen without causing persistent urethral irritation with repeated urethral catheterization. Cystostomy tubes are not meant to be a long‐term solution as they are fragile catheters, therefore care needs to be taken to ensure proper use and management to best avoid complications, which were often iatrogenic of dog induced dislodgement from failure to wear a protective collar or lack of dog oversight by caregivers.

While the authors feel the use of percutaneous cystostomy tubes is ultimately beneficial in cases of FOO, refractory or not, the complication rates are significant. Percutaneous cystostomy tubes should only be placed by veterinarians with specialized training and clients that can be trusted to understand the fragility of the catheters and need for oversight or protective collars, since all dislodgement events happened when the dogs were unsupervised.

Major limitations in this study are owing to its retrospective nature including inability to standardize diagnosis, treatment, medication dosing, timing of intervention, and follow‐up times. In this study, a diagnosis of FOO was made based off consistent clinical signs and imaging including contrast cystourethrogram or cystourethroscopy to rule out MOO. However, none of the included dogs had a documented PVRV which is part of a newer standard of diagnostic criteria. Additionally, the small number of cases makes statistical comparisons difficult.

Overall, dogs with cystostomy tubes had good to excellent outcomes regardless of whether they were refractory to medical management or had complications related to their tubes. These results were considered superior to the group of dogs that did not have a cystostomy tube as part of their management plan, supporting the consideration to discuss cystostomy tubes with clients at the time of diagnosis of FOO and the potential for a good to excellent long‐term outcome. To further define success, a prospective study evaluating a standardized medical management regimen before consideration of cystostomy tube placement would be ideal to truly understand success and failure rates.

## CONFLICT OF INTEREST DECLARATION

Authors declare no conflict of interest.

## OFF‐LABEL ANTIMICROBIAL DECLARATION

Authors declare no off‐label use of antimicrobials.

## INSTITUTIONAL ANIMAL CARE AND USE COMMITTEE (IACUC) OR OTHER APPROVAL DECLARATION

Authors declare no IACUC or other approval was needed.

## HUMAN ETHICS APPROVAL DECLARATION

Authors declare human ethics approval was not needed for this study.
